# Association of Physician Characteristics With Early Adoption of Virtual Health Care

**DOI:** 10.1001/jamanetworkopen.2021.41625

**Published:** 2021-12-30

**Authors:** Kori S. Zachrison, Zhiyu Yan, Margaret E. Samuels-Kalow, Adam Licurse, Gianna Zuccotti, Lee H. Schwamm

**Affiliations:** 1 Department of Emergency Medicine, Massachusetts General Hospital, Boston; 2 Department of Neurology, Massachusetts General Hospital, Boston; 3 Mass General Brigham, Somerville, Massachusetts; 4 Department of Medicine, Brigham and Women’s Hospital, Boston, Massachusetts; 5 Division of Infectious Disease, Brigham and Women’s Hospital, Boston, Massachusetts

## Abstract

**Importance:**

The rapid transition to virtual health care has depended on physician and patient abilities to adopt new technology and workflows. Physicians transitioning more slowly or not at all could result in access challenges for their patients.

**Objective:**

To identify physician characteristics associated with the transition to virtual health care in a large regional health care system.

**Design, Setting, and Participants:**

This retrospective cross-sectional study uses administrative health system databases to analyze data from all 3473 physicians providing ambulatory care through a large New England health care system, which includes 12 hospitals and their ambulatory practices, from October 1, 2019, through December 31, 2020.

**Exposures:**

Physicians characterized based on gender, popularized generational demographic cohort (Silent Generation, born 1928-1945; Baby Boomers, born 1946-1964; Generation X, born 1965-1980; and Millennials, born 1981-1996), specialty (behavioral health, primary care, medical, and surgical), and hospital affiliation as well as selected patient characteristics (number of visits and proportion of patients with self-pay or Medicaid insurance, aged 65 years or older, preference for speaking a language other than English, from a racial or ethnic minority group, and with an active patient portal).

**Main Outcomes and Measures:**

Early adoption of virtual health care. Bivariate comparisons were made, and regression modeling was used to examine characteristics associated with the likelihood of early adoption of virtual health care.

**Results:**

Of 3473 physicians conducting ambulatory visits during the study period, 1624 (46.8%) were women, 83 (2.4%) were in the Silent Generation, 994 (28.6%) were Baby Boomers, 1637 (47.1%) were in Generation X, and 759 (21.9%) were Millennials. There were 1649 physicians (47.5%) in medical specialties, 749 physicians (21.6%) in surgical specialties, and 248 physicians (7.1%) in behavioral health. After accounting for other characteristics, female (odds ratio [OR], 1.23; 95% CI, 1.06-1.44), behavioral health (OR, 2.92; 95% CI, 2.11-4.04), and primary care (OR, 1.69; 95% CI, 1.36-2.09) physicians had greater odds of being early adopters, and physicians in the Silent Generation (OR, 0.39, 95% CI, 0.24-0.65) and in surgical specialties (OR, 0.46; 95% CI, 0.38-0.57) were less likely to be early adopters. Patient characteristics were less strongly associated with physician adoption.

**Conclusions and Relevance:**

In this cross-sectional study, there was physician-level variation in the adoption of virtual health care, with female, primary care, and behavioral health physicians in this system most likely to lead the transformation to virtual health care.

## Key Points

**Question:**

What physician characteristics were associated with early and sustained adoption of virtual health care?

**Findings:**

In a cross-sectional study of 3473 physicians in a large regional health care system, more than 94% transitioned to include virtual health care in their practice by December 2020. Female, behavioral health, and primary care physicians were more likely to be early adopters, and physicians born between 1928 and 1945 (Silent Generation) and in surgical specialties were less likely to be early adopters.

**Meaning:**

Although most physicians in the system adopted virtual health care in 2020, female, primary care, and behavioral health physicians were most likely to play an important role as early adopters.

## Teaser

This cross-sectional study uses administrative health system databases to identify physician characteristics associated with early transition to virtual health care among all physicians providing ambulatory care through a large New England health care system from October 2019 through December 2020.

## Introduction

The transition to telehealth during the COVID-19 pandemic was critical to continue to provide health care to patients.^1,2,3,4^ This change has also enabled major shifts in access to care, with increased safety and convenience for patients, minimizing potential exposures, eliminating travel requirements, and reducing need for time off from work to complete a visit.^5,6,7^ Correspondingly, physicians have made major shifts in their approaches to the delivery of care to make this happen. Yet some physicians may have been nimbler than others in making the transition, potentially influencing patients’ experience of physician access and availability.

Prior work in health information technology has found earlier adoption of technology among physicians affiliated with larger health systems and sharing resources with other practices.^8,9^ There has been less work done to understand the characteristics of physicians who adopt virtual health care delivery during the pandemic. Most researchers have assumed that the transition occurred “all at once” or at least all at once within a given system. Yet, if some physicians have been slower or even dilatory with respect to the transition to virtual care, this may lead to their patients unintentionally being left behind. Physicians’ lack of buy-in and resistance to change have been previously cited as barriers to adoption of video consultation.^10^ In addition, a perceived low level of digital health literacy, economic hardship, or limited English proficiency may lead some physicians to refrain from performing virtual visits with certain patient groups. Perceived convenience may also play a role, for example, if the need for language interpretation was seen as a potential or real barrier.^11^

To inform this important question in health care access, we sought to understand the physician characteristics that were associated with the transition to virtual care in a large, regional 12-hospital health system. Our primary objective was to identify physician characteristics associated with early and sustained adoption of virtual health care. We hypothesized that adoption would vary by generational demographic cohort.

## Methods

### Study Setting, Data Sources, and Sample

The study was conducted in Mass General Brigham, a large New England health care system that includes 12 hospitals and their outpatient practice sites. We used our organizational master credentialing database to identify all credentialed physicians during the October 1, 2019, through December 31, 2020, study period. We then used encounter-level data from the enterprisewide integrated electronic health record (Epic Systems) to identify all ambulatory visits associated with physicians in our sample. We excluded physicians without at least 1 virtual or in-person visit prior to March 15, 2020, and without at least 1 virtual or in-person visit after March 15, 2020. This was the date of the public health emergency declaration in our state (Massachusetts) and also the time when the major virtual health care transition occurred in our system. Patterns of adoption by physicians without visits both prior to and after that date would not be readily categorized. Similar to many large academic health systems, the transition to virtual care in our system was scaled up rapidly at the time of the public health emergency declaration. To reduce viral transmission and to preserve personal protective equipment, in accordance with local public health emergency regulations, an ambulatory visit capability was rapidly deployed throughout our health care system and made available to all sites simultaneously. However, there was an independent approach for each of the 12 hospitals, their affiliated outpatient practice sites, and the many physicians at those sites that contributed to variation in the pace and ease of transition. The technology, software support, and platforms were enabled centrally all at once, but the individual sites were provided implementation support at their own discretion. This report followed the Strengthening the Reporting of Observational Studies in Epidemiology (STROBE) reporting guideline for cross-sectional studies. The institutional review board of Mass General Brigham, Boston, Massachusetts, approved the study and granted an exemption from the requirement for obtaining informed consent.

### Variables of Interest

The physician characteristics obtained from the credentialing database included age, gender, and years since medical school graduation. We used year of birth to categorize physicians by popularized generational demographic cohort into the Silent Generation (1928-1945), Baby Boomers (1946-1964), Generation X (1965-1980), and Millennials (1981-1996) using the Brookings Institution classification.^12^

Physician characteristics identified in the encounter-level database included specialty and hospital affiliation. We used specialty to group physicians into behavioral health, primary care, medical, or surgical categories. We used hospital affiliation to categorize physicians dichotomously based on affiliation with one of the major teaching hospitals in our system. We also identified a set of characteristics associated with the patients treated by each physician (in person and virtually) during the period of interest. These variables were aggregated at the physician level and used for adjustment, and they included the number of unique patients with whom they had any type of visit during the study period, the proportion of patients with self-pay or Medicaid insurance, the proportion of patients 65 years of age or older, the proportion of patients who prefer speaking a language other than English, the proportion of patients from a racial or ethnic minority group, and the digital literacy of their patients defined by the proportion of patients with an activated patient portal. Many of these physician-level variables (eg, the proportion of patients from a racial or ethnic minority group) were included to specifically examine whether physician characteristics were associated with differences in access to virtual health care for groups of patients who have traditionally experienced barriers in access to care.

### Outcome of Interest

The adoption of virtual health care by physicians was defined based on the date their first virtual visit was completed. In-person vs virtual visits were identified using modifier codes associated with each visit. We analyzed all visit and encounter types classified as telemedicine or virtual health care, and we used billing modifiers to identify whether visits were conducted either by telemedicine (GT modifier) or telephone (GPH, a custom modifier that we built for this purpose). This enabled us to exclude any customary telephone calls from the virtual visit analysis.

We used our data to derive categories of adoption. We categorized physicians as innovators (those with virtual visits before March 15, 2020, the start of the pandemic in Boston), early adopters (those adopting during the week starting March 15, 2020), majority (those adopting on March 22, 2020 or later), and persistent nonadopters (no adoption through December 31, 2020). Although the majority group in the technological adoption schematic typically represents the true majority of a bell-shaped curve, the circumstances of the pandemic so rapidly catalyzed and compressed adoption that the adoption curve in our data is heavily skewed. Thus, we used qualitative definitions to guide our data-driven categorizations. Namely, we classified physicians as “innovators” when they were using the technology when it was truly cutting edge, prior to the pandemic-catalyzed adoption. We classified physicians as “early adopters” when they were using the technology after the perceived benefit, adopting it in the first week after the public health declaration, when platforms and access to technical support were variable. We classified physicians as “majority” when they were using the technology when there was a productivity gain, adopting it after the first week of the transition, when hospitals and the health system had been able to scaffold greater technological support for physicians in the transition and supported submission of claims for reimbursement.

### Statistical Analysis

We used standard descriptive statistics to characterize the sample of physicians overall and by adoption group. We used standardized mean differences (SMDs) to identify meaningful differences between groups, comparing across adoption categories and also comparing innovators and early adopters vs all others, and considered an SMD less than 0.1 as a cutoff for acceptable balance. We also used χ^2^ tests of independence to evaluate whether the distribution of virtual health care adoption varied by gender, by generational cohort, or by specialty group, with *P* values calculated using Monte Carlo simulation with 5000 replicates and a statistical significance level of *P* < .05. Finally, we used a logistic regression model to identify characteristics associated with being an innovator or early adopter. The variables of interest included in the model were generational demographic cohort, gender, major teaching hospital affiliation, specialty class (medical, surgical, or behavioral health), and characteristics of physicians’ patients (total number of unique patients seen during the study period, percentage of patients with self-pay or Medicaid insurance, percentage of patients 65 years of age or older, percentage of patients who spoke little English, percentage of patients from a racial or ethnic minority group, and percentage of patients with an activated portal). We excluded observations with missing data (0.5% for missing specialty) from the model. We also tested for interactions between gender and generational demographic cohort, gender and patient volume, gender and specialty class, and generational demographic cohort and specialty class. We used Wald tests to evaluate interaction terms in the regression models and determined whether the 95% Wald CIs contained 0 (ie, if the estimate was significantly different from 0). Because none of these interactions were significant, they were dropped for ease of interpretation. We also performed a logistic regression model to examine factors independently associated with the odds of persistent nonadoption, using the same variables of interest and testing the same interactions. Finally, we performed a sensitivity analysis using age-based cutoffs in place of the generational demographic cohorts. All analyses were conducted with R, version 4.0.3 (R Foundation for Statistical Computing).

## Results

### Physician Characteristics

We identified 3473 physicians in our health system who conducted any outpatient ambulatory virtual or in-person visits both before and after March 15, 2020 (Table 1). The mean (SD) physician age was 50.5 (11.6) years, 1624 physicians (46.8%) were women, and 1849 physicians (53.2%) were men (eFigure 3 in the Supplement). Female physicians were younger than male physicians (mean age, 47.8 years vs 52.9 years; SMD, 0.5). When classified by generational demographic cohort, 83 physicians (2.4%) were in the Silent Generation, 994 physicians (28.6%) were Baby Boomers, 1637 physicians (47.1%) were in Generation X, and 759 physicians (21.9%) were Millennials. Most physicians (2289 [65.9%]) were affiliated with 1 of our 2 academic hospitals. There were 807 physicians (23.2%) in primary care, 1649 physicians (47.5%) in medical specialties, 749 physicians (21.6%) in surgical specialties, 248 physicians (7.1%) in behavioral health, and 20 physicians (0.6%) with missing specialty information.

**Table 1.** Characteristics of Physicians and Physicians’ Patients

Characteristic	Innovator or early adopter (n = 2040)	Majority (n = 1237)	Nonadopter (n = 196)	Overall (n = 3473)	Comparison across groups, SMD
Physicians					
Age, mean (SD), y	50.0 (11.0)	50.9 (12.4)	53.0 (12.2)	50.5 (11.6)	0.17
Demographic cohort, No. (%)					
Silent Generation	29 (34.9)	48 (57.8)	6 (7.2)	83 (100)	0.20
Baby Boomer	571 (57.4)	354 (35.6)	69 (6.9)	994 (100)	
Generation X	997 (60.9)	550 (33.6)	90 (5.5)	1637 (100)	
Millennial	443 (58.4)	285 (37.5)	31 (4.1)	759 (100)	
Gender, No. (%)					
Female	1044 (51.2)	514 (41.6)	66 (33.7)	1624 (46.8)	0.24
Male	996 (48.8)	723 (58.4)	130 (66.3)	1849 (53.2)	
Time since medical school graduation, y					
Mean (SD)	21.8 (11.4)	22.6 (12.6)	24.6 (13.2)	22.2 (12.0)	0.15
Missing data, No. (%)	262 (12.8)	155 (12.5)	45 (23.0)	462 (13.3)	
Major teaching hospital affiliation, No. (%)					
No	702 (59.3)	389 (32.9)	93 (7.9)	1184 (100)	0.22
Yes	1338 (58.5)	848 (37.0)	103 (4.5)	2289 (100)	
Specialty group, No. (%)					
Behavioral health	177 (71.4)	69 (27.8)	2 (0.8)	248 (100)	0.48
Primary care	599 (74.2)	185 (22.9)	23 (2.9)	807 (100)	
Medical	924 (56.0)	602 (35.4)	123 (7.5)	1649 (100)	
Surgical	331 (44.2)	370 (49.4)	48 (6.4)	749 (100)	
Missing	9 (45)	11 (55)	0	20 (100)	
Patient characteristics at the physician level, mean (SD)					
Patient volume	697 (534)	511 (553)	247 (513)	606 (554)	0.57
% With self-pay or Medicaid insurance	12.0 (11.2)	11.8 (11.1)	13.4 (16.0)	12.0 (11.5)	0.08
% Patients aged ≥65 y	33.8 (21.5)	36.3 (22.5)	35.8 (24.2)	34.8 (22.0)	0.07
% Preferring a non-English language	7.3 (8.3)	7.9 (8.0)	8.5 (9.1)	7.6 (8.3)	0.09
% From a racial or ethnic minority group	21.2 (14.3)	22.2 (15.0)	22.2 (18.0)	21.6 (14.8)	0.05
% With activated portal	76.5 (14.2)	72.1 (15.2)	59.4 (21.6)	73.9 (15.6)	0.64
% of Audio-only visits	34.8 (24.6)	42.1 (32.2)	NA^a^	37.5 (27.9)	0.25

Abbreviations: NA, not applicable; SMD, standardized mean difference.

^a^ Proportion of virtual visits that were audio only was not calculated among persistent nonadopters because these physicians did not have any virtual visits.

### Characteristics of Physicians’ Patients

The median number of unique patients per physician was 450 (IQR, 170-906). We described physicians by the characteristics of their patients. The mean (SD) proportion of patients with self-pay or with Medicaid insurance who were seen by a physician was 12.0% (11.5%). The mean (SD) proportion of patients who were 65 years of age or older was 34.8% (22.0%). The mean (SD) proportion of patients who preferred speak a language other than English was 7.6% (8.3%), and the mean (SD) proportion of patients from a racial or ethnic minority group was 21.6% (14.8%).

### Characteristics of Physicians’ Virtual Health Care Delivery

**Figure 1.**
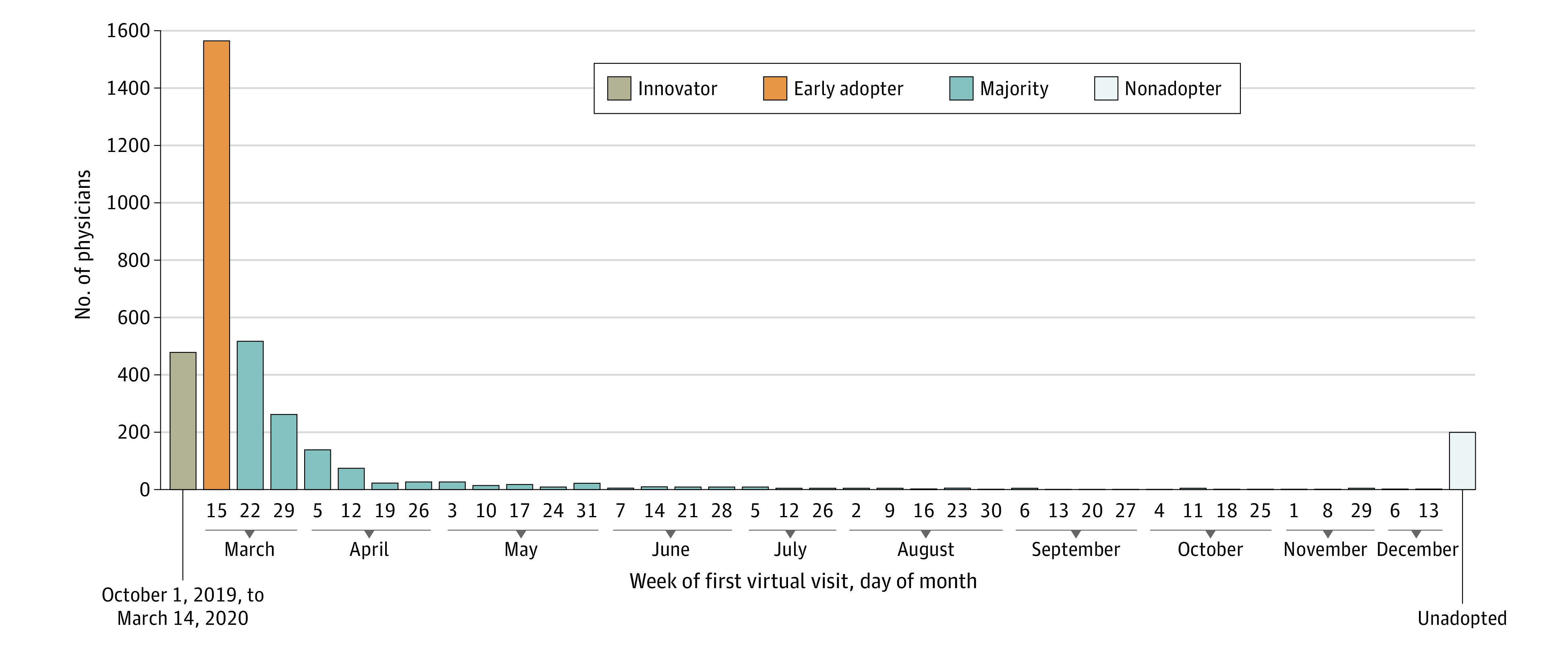
Timing of Virtual Care Adoption by 3473 Physicians All dates are for 2020 except for the first date range.

**Figure 2.**
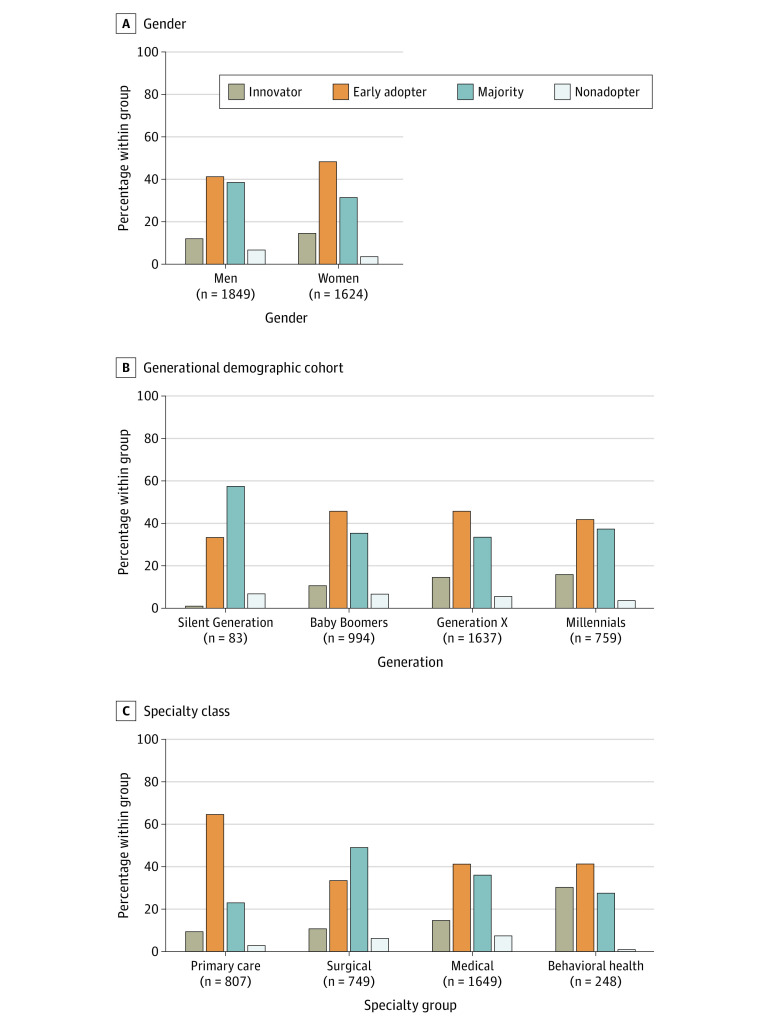
Category of Virtual Adoption by Gender, Generational Demographic Cohort, and Specialty Class Values in parentheses indicate total number of physicians within the group. Distribution of the adoption of virtual heath care was significantly different among genders, demographic age cohorts, and specialty classes.

Of 3473 physicians, 478 (13.8%) were innovators (adopted virtual care prior to March 15, 2020), 1562 (45.0%) were early adopters (adopted during the week starting March, 15, 2020), 1237 (35.6%) were in the majority (adopting after the week starting March 15, 2020), and 196 (5.6%) were persistent nonadopters and had not used virtual care as of December 31, 2020 (Figure 1). We also stratified virtual adoption category by gender, generational demographic cohort, and specialty class. Younger generations had larger proportions of innovators and early adopters. When examined by specialty class, behavioral health and primary care had the largest proportion of innovators (behavioral health, 75 of 248 [30.2%]; primary care, 76 of 807 [9.4%]) and early adopters (behavioral health, 102 of 248 [41.1%]; primary care, 523 of 807 [64.8%]), followed by medical specialties (innovators, 244 of 1649 [14.8%]; early adopters, 680 of 1649 [41.2%]), with the lowest among surgical specialties (innovators, 80 of 749 [10.7%]; early adopters, 251 of 749 [33.5%]) (Figure 2).

**Figure 3.**
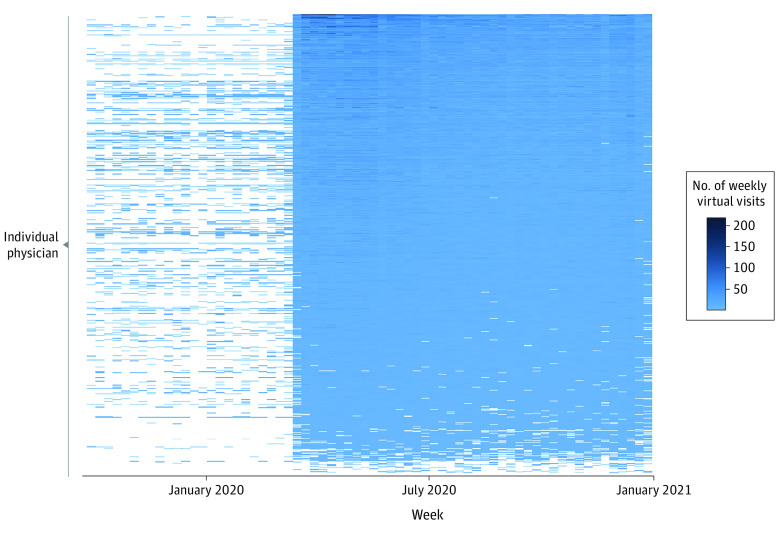
Intensity of Virtual Visit Use per Individual Physician by Week Physicians with at least 1 virtual visit during the study period are included. Each row represents a unique physician. For each physician, the number of virtual visits conducted each week of the study period is represented by shading.

Of 3277 physicians providing virtual health care, 2863 (87.4%) had a mean of at least 1 virtual visit per week from March 15 through December 31, 2020, with very few of the full sample (49 [1.5%]) performing only 1 visit or performing 1 day of virtual visits (62 [1.9%]). Of 478 innovators, 476 (99.6%) provided ongoing virtual care, and physicians in this group had a mean (SD) of 12 (8) virtual visits per week from March 15 through 31, 2020. Of all 3473 physicians, 2863 (82.4%) had a mean of at least 1 virtual visit per week from adoption through the end of the study period. In addition, most physicians continued to provide virtual care even after returning to relatively normal operating procedures, with 2640 (80.6% of all virtual care–providing physicians) performing at least 1 virtual visit per week from September 15 through December 31, 2020 (Figure 3).

Among 3277 physicians providing virtual health care, the median proportion of audio-only visits conducted by each physician was 31.8% (IQR, 15.5%-53.4%). This proportion decreased as age demographic cohort decreased, with a median of 41.5% (IQR, 19.9%-70.4%) among physicians in the Silent Generation, 35.6% (IQR, 18.4%-62.0%) among Baby Boomers, 30.2% (IQR, 15.2%-50.0%) among Generation X, and 28.5% (IQR, 13.6%-51.6%) among Millennials. Histograms of the percentage of audio-only visits to all virtual visits stratified by generational demographic cohort are presented in eFigure 1 in the Supplement.

### Physician Characteristics Associated With Innovator or Early Adopter Status

In bivariate comparisons, innovators and early adopters were more often female (1044 of 2040 [51.2%] of early adopters and innovators were female vs 580 of 1433 [40.5%] of all others; SMD, 0.22) and from behavioral health and primary care (177 of 2031 [8.7% of early adopters and innovators] from behavioral health, 599 of 2031 [29.5%] from primary care, 924 of 2031 [45.5%] from medical specialties, and 331 of 2031 [16.3%] from surgical specialties); all other physicians were from behavioral health (71 of 1422 [5.0%]), primary care (208 of 1422 [14.6%]), medical specialties (725 of 1422 [51.0%]), and surgical specialties (418 of 1422 [29.4%]) (SMD, 0.46). Innovators and early adopters had a higher patient volume (mean number of patients, 697 vs 475; SMD, 0.41), and a greater proportion of patients with an activated patient portal (mean proportion, 76.5% vs 70.3%; SMD, 0.39). Other patient characteristics did not vary between early adopters and innovators vs other physicians, including similar proportions of patients with self-pay and Medicaid insurance, 65 years of age or older, preferring to speak a language other than English, and from a racial or ethnic minority group (eTable 1 in the Supplement).

After accounting for other characteristics in logistic regression modeling, we found that the physician characteristics associated with the likelihood of innovator or early adopter status were generational demographic cohort (odds ratio [OR], 0.39 for Silent Generation relative to Generation X; 95% CI, 0.24-0.65), female gender (OR, 1.23; 95% CI, 1.06-1.44), specialty class (relative to medical specialties, OR for primary care 1.69 [95% CI, 1.36-2.09]; OR for surgical, 0.46 [95% CI, 0.38-0.57]; and OR for behavioral health, 2.92; [95% CI, 2.11-4.04]), physicians’ number of patients (OR, 1.01 per 10-patient increase; 95% CI, 1.01-1.01), physicians’ proportion of patients from a racial or ethnic minority (OR, 0.94 per 5% increase; 95% CI, 0.90-0.98), physicians’ proportion of patients preferring to speak a language other than English (OR, 1.09 per 5% increase; 95% CI, 1.01-1.17), and physicians’ percentage of patients with an activated portal (OR, 1.18 per 5% increase; 95% CI, 1.14-1.21) (Table 2). The results were similar when we used age categories in place of generational demographic cohorts (eTable 2 and eFigure 2 in the Supplement). The results of the models testing interaction terms are given in eTable 3 and eTable 4 the Supplement.

**Table 2.** Physician Characteristics Associated With Likelihood of Early Adoption of Virtual Health Care (Innovator or Early Adopter Status) in Logistic Regression Modeling

Characteristic	Odds Ratio (95% CI)	Mean Marginal Effect (95% CI)
Demographic cohort		
Silent Generation	0.39 (0.24 to 0.65)	–0.20 (–0.30 to –0.09)
Baby Boomer	0.88 (0.74 to 1.05)	–0.03 (–0.06 to 0.01)
Generation X	1 [Reference]	1 [Reference]
Millennial	0.92 (0.76 to 1.12)	–0.02 (–0.06 to 0.02)
Gender		
Male	1 [Reference]	1 [Reference]
Female	1.23 (1.06 to 1.44)	0.04 (0.01 to 0.08)
Major teaching hospital affiliation	0.93 (0.78 to 1.12)	–0.01 (–0.05 to 0.02)
Specialty class		
Behavioral health	2.92 (2.11 to 4.04)	0.20 (0.15 to 0.25)
Primary care	1.69 (1.36 to 2.09)	0.11 (0.06 to 0.15)
Medical	1 [Reference]	1 [Reference]
Surgical	0.46 (0.38 to 0.57)	–0.17 (–0.21 to –0.13)
Characteristic of physicians’ patients		
Total No. of patients (per 10-patient increase)	1.01 (1.01 to 1.01)	0.002 (0.002 to 0.003)
% of Patients self-pay or Medicaid insurance (per 5% increase)	1.05 (0.99 to 1.04)	0.01 (0.00 to 0.02)
% of Patients aged >65 y (per 5% increase)	1.01 (0.99 to 1.04)	0.00 (0.00 to 0.01)
% of Patients non-English speaking (per 5% increase)	1.09 (1.01 to 1.17)	0.02 (0.00 to 0.03)
% of Patients from a racial or ethnic minority group (per 5% increase)	0.94 (0.90 to 0.98)	–0.01 (–0.02 to 0.00)
% of Patients with activated portal (per 5% increase)	1.18 (1.14 to 1.21)	0.03 (0.03 to 0.04)

We also examined physician characteristics associated with persistent nonadoption of virtual visits, with results presented in eTable 5 in the Supplement. Physicians who were Millennial (OR, 0.61; 95% CI, 0.39-0.97), were women (OR, 0.67; 95% CI, 0.47-0.95), had a major teaching hospital affiliation (OR, 0.51; 95% CI, 0.35-0.73), were in primary care (OR, 0.54; 95% CI, 0.31-0.96) or behavioral health (OR, 0.02; 95% CI, 0.01-0.10), had higher patient volume (OR per 10-patient increase, 0.97; 95% CI, 0.96-0.98), or had a greater percentage of patients with an activated portal (OR, per 5% increase 0.77; 95% CI, 0.74-0.81) had decreased odds of persistent nonadoption after accounting for other characteristics (eTable 2 in the Supplement).

## Discussion

In this cross-sectional study of more than 3400 physicians providing ambulatory care in our large regional health system, the overwhelming majority of physicians (94.4%) transitioned to include virtual health care in their practice by the end of 2020. There were minor differences by generational demographic cohort, and female physicians and behavioral health physicians were the most likely to be early adopters.

We are not aware of prior reports describing earlier adoption of virtual health care by female physicians relative to their male counterparts. We initially hypothesized that this may have been associated with different age distributions within our system—with greater representation of women among the younger cohorts of physicians. However, we tested an interaction to examine whether the association between gender and earlier adoption may have been influenced by age or generational demographic cohort differences, and we did not find a significant interaction. There are many other potential explanations for the higher rates of early adoption among female physicians relative to male physicians. Although we did not collect data to explore why some physicians did or did not adopt virtual health care, there are several plausible explanations. For example, the toll of the pandemic on women in caregiving roles has been well described, and this group may have found that virtual care provided a flexible solution that enabled them to balance or maintain their many roles. Although many things happened simultaneously during the early weeks of the pandemic, we note that the peak of early adoption coincided with the state-mandated school closure date of March 16, 2020.^13^

An alternative potential explanation may be found in communication practices. Female physicians have previously been shown to have more patient-centered communication and to spend more time with their patients,^14,15^ and it is possible that their earlier transitions to virtual health care were a result of being more responsive to their perceptions of their patients’ needs with rapid changes early in the pandemic.

When we examined medical specialty type, we found that the physician factor most strongly associated with likelihood of early adoption of virtual health care was practicing in behavioral health, followed by primary care. There are a number of potential explanations for this association. Behavioral health visits may be more amenable to virtual care given that they are typically less reliant on an in-person physical examination or procedure. It is also likely that patients with behavioral health needs had particular challenges forgoing medical care when in-person visits were restricted at the onset of the public health emergency, and thus these challenges motivated their physicians to make a rapid transition to virtual care.

We had hypothesized that physicians’ generational demographic cohort would be an important factor associated with the likelihood of early adoption. Telehealth shares similarities with other technologies on smartphones, tablets, and computers, and preexisting experience and confidence may have influenced attitudes and adoption,^16^ and physicians’ experience with technology has previously been identified as a barrier.^17^ Yet the only generational association that we noted was that the small number of physicians in our system from the Silent Generation were less likely to be early adopters. However, more than 90% of those older physicians ultimately did transition to providing virtual health care at some point in 2020, and membership in the Silent Generation was not associated with persistent nonadoption. When considering physicians in the Baby Boomer generation, we found that they were as likely to be early adopters as physicians in the Generation X or Millennial groups. Although possibly initially surprising, this is not inconsistent with general technology trends. Millennials do lead in adoption and use of technology, but Baby Boomers, in particular, have shown rapidly increasing rates of technology adoption.^18^

Physicians’ transition to virtual health care may also be associated with their knowledge of their patient panels. We found that physicians with higher proportions of patients with activated portals were more likely to be early adopters. However, there is a chicken-egg question at play here as well, given that more technologically leaning physicians may have previously encouraged portal activation for their patients to a greater extent. We had hypothesized that physicians may also show bias associated with their patient panels and their capabilities. Reassuringly, the proportion of patients 65 years or older who a physician cared for was not associated with odds of earlier adoption, nor was the proportion of patients with self-pay or Medicaid insurance. Interestingly, we found slightly increased odds of early adoption with increasing percentage of patients who preferred speaking a language other than English but slightly decreased odds of early adoption with increasing percentage of patients from a racial or ethnic minority group. Further work examining the interaction between physicians’ patient panels and adoption of virtual health care will be critical to ensure equitable access to care across patient groups. Better understanding the availability and role of audio-only virtual visits will also be important considerations in the aim for improved digital health equity.

Overall, this study did not explore factors that are subject to ready influence in terms of promoting adoption, as the exposure to COVID-19 was immediate and intense and simultaneously affected all physicians. However, several factors from our experience are worth noting and may inform future work in technology adoption. First, the presence of a unified electronic health record that was linked to virtual care delivery enabled us to scale the implementation across all practices simultaneously. Second, the presence of reimbursement and the need for patient care access to be preserved created both missional and financial alignment for physicians and the health system organization. Third, the technology necessary to enable virtual video visits was consumer grade and widely available, using physicians’ personal computers augmented by external web cameras and headsets if needed. Lastly, the pandemic unified health care workers and galvanized a humanitarian response and esprit de corps to ensure that health care could continue to be delivered. These structural factors likely contributed to massive adoption and may provide some guidance for future technology adoption strategies.

### Limitations

Our study has limitations. These findings are from a single regional health system in the Northeast United States and may not be generalizable to other settings. In addition, our analysis was limited to administrative data, and there may be other important confounders (such as beliefs, attitudes, or prior experience) associated with physicians’ adoption of virtual health care that we were unable to consider. Owing to the very rapid occurrence of adoption, there was also little room for separation of traits across categories of adoption, which may have limited our ability to distinguish between groups. Finally, with various training programs and academically affiliated faculty, physician turnover is common in our system, and there may have been an influx or outflow of physicians during the study period that may have influenced our results.

## Conclusions

The findings of this cross-sectional analysis highlight the variation in the adoption of virtual health care at the physician level, but ultimately, the vast majority of physicians in our health system adopted virtual care in 2020. Variation in adoption was associated with physician gender, specialty type, and generational differences, as well as with characteristics of physicians’ patients. Female physicians and behavioral health physicians played an important role as early adopters in our system. Future work should examine how much variation in patients’ transitions to virtual health care is explained by these physician-level differences, including whether characteristics of the patient-physician dyad are influential.

## Notes

Supplement. **eFigure 1.** Proportion of Virtual Visits That Were Audio-Only, by Demographic Cohort **eFigure 2.** Category of Virtual Adoption by Age Category **eFigure 3.** Comparison of Age Distribution Between Genders **eTable 1.** Comparison of Innovators/Early Adopters vs Other Physicians **eTable 2.** Physician Characteristics Associated With Likelihood of Early Virtual Care Adoption (Innovator or Early Adopter Status) in Logistic Regression Modeling, Using Age in Place of Demographic Cohort Categories **eTable 3.** Physician Characteristics Associated With Likelihood of Early Virtual Care Adoption (Innovator or Early Adopter Status) in Logistic Regression Modeling, Including Interaction for Demographic Cohort and Specialty **eTable 4.** Physician Characteristics Associated with Likelihood of Early Virtual Care Adoption (Innovator or Early Adopter Status) in Logistic Regression Modeling, Including Interaction Terms for Gender and Generation and for Gender and Specialty Class **eTable 5.** Physician Characteristics Associated with Likelihood of Persistent Nonadoption of Virtual Care in Logistic Regression Modeling
