# Supplementary material for: Association of Physician Characteristics With Early Adoption of Virtual Health Care
Source: JAMA Netw Open. 2021 Dec 30;4(12):e2141625. doi: 10.1001/jamanetworkopen.2021.41625 (PMC8719243; doi:10.1001/jamanetworkopen.2021.41625)
Supplement: Supplement. — eFigure 1. Proportion of Virtual Visits That Were Audio-Only, by Demographic Cohort eFigure 2. Category of Virtual Adoption by Age Category eFigure 3. Comparison of Age Distribution Between Genders eTable 1. Comparison of Innovators/Early Adopters vs Other Physicians eTable 2. Physician Characteristics Associated With Likelihood of Early Virtual Care Adoption (Innovator or Early Adopter Status) in Logistic Regression Modeling, Using Age in Place of Demographic Cohort Categories eTable 3. Physician Characteristics Associated With Likelihood of Early Virtual Care Adoption (Innovator or Early Adopter Status) in Logistic Regression Modeling, Including Interaction for Demographic Cohort and Specialty eTable 4. Physician Characteristics Associated with Likelihood of Early Virtual Care Adoption (Innovator or Early Adopter Status) in Logistic Regression Modeling, Including Interaction Terms for Gender and Generation and for Gender and Specialty Class eTable 5. Physician Characteristics Associated with Likelihood of Persistent Nonadoption of Virtual Care in Logistic Regression Modeling [file jamanetwopen-e2141625-s001.pdf]

Supplementary Online Content

Zachrison KS, Yan Z, Samuels-Kalow ME, Licurse A, Zuccotti G, Schwamm LH. Association of physician characteristics with early adoption of virtual health care. *JAMA Netw Open*. 2021;4(12):e2141625. doi:10.1001/jamanetworkopen.2021.41625

eFigure 1. Proportion of Virtual Visits That Were Audio-Only, by Demographic Cohort
eFigure 2. Category of Virtual Adoption by Age Category
eFigure 3. Comparison of Age Distribution Between Genders
eTable 1. Comparison of Innovators/Early Adopters vs Other Physicians
eTable 2. Physician Characteristics Associated With Likelihood of Early Virtual Care Adoption (Innovator or Early Adopter Status) in Logistic Regression Modeling, Using Age in Place of Demographic Cohort Categories
eTable 3. Physician Characteristics Associated With Likelihood of Early Virtual Care Adoption (Innovator or Early Adopter Status) in Logistic Regression Modeling, Including Interaction for Demographic Cohort and Specialty
eTable 4. Physician Characteristics Associated with Likelihood of Early Virtual Care Adoption (Innovator or Early Adopter Status) in Logistic Regression Modeling, Including Interaction Terms for Gender and Generation and for Gender and Specialty Class
eTable 5. Physician Characteristics Associated with Likelihood of Persistent Nonadoption of Virtual Care in Logistic Regression Modeling

This supplementary material has been provided by the authors to give readers additional information about their work.

eFigure 1. Proportion of Virtual Visits That Were Audio-Only, by Demographic Cohort

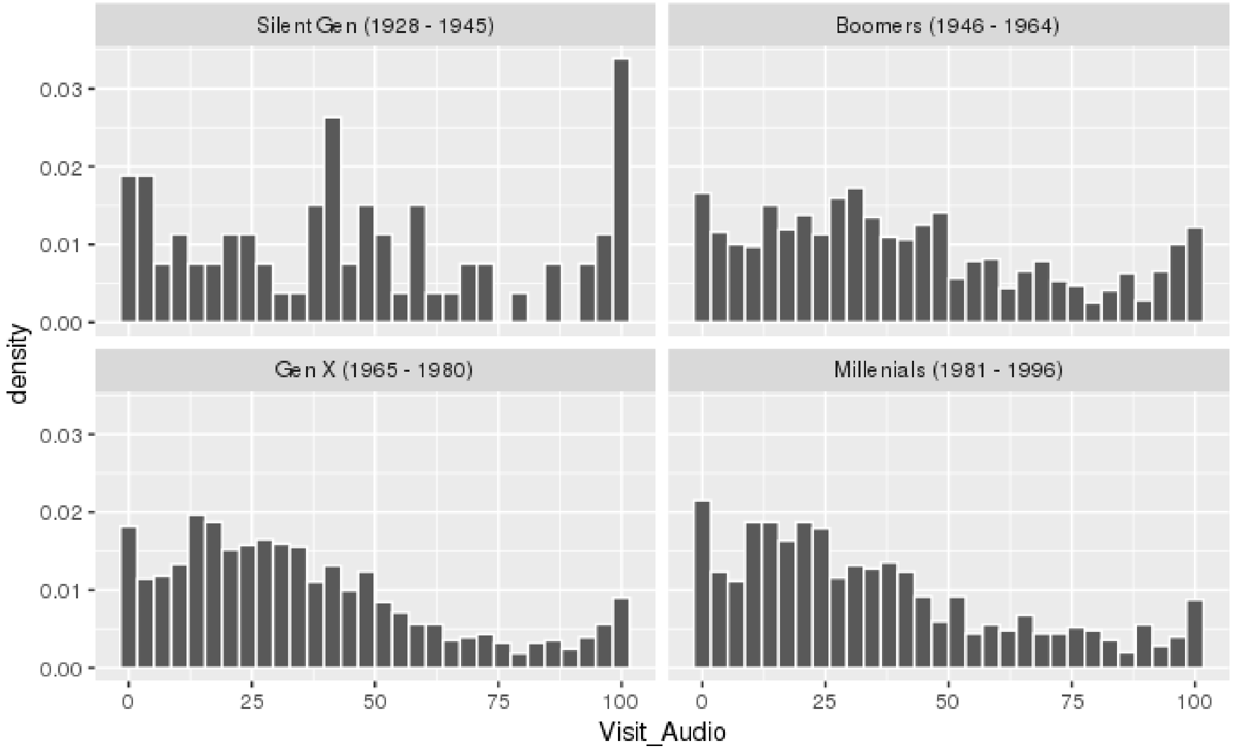

© 2021 Zachrison KS et al. *JAMA Network Open*.

eFigure 2. Category of Virtual Adoption by Age Category

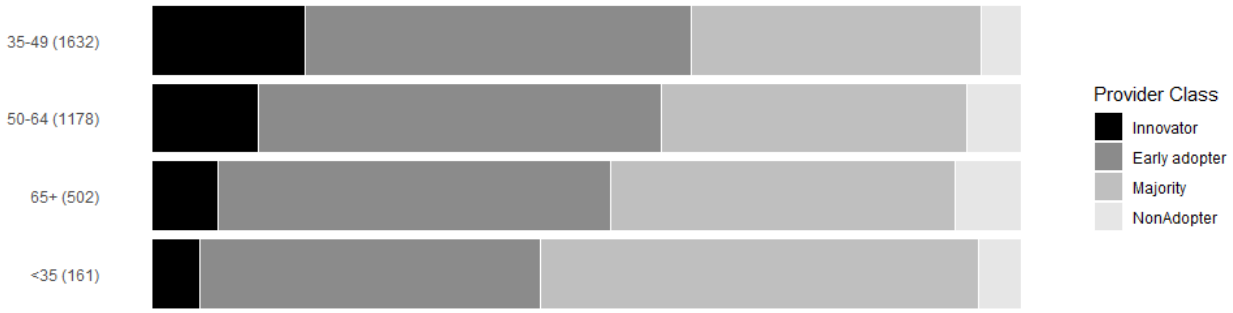

© 2021 Zachrison KS et al. *JAMA Network Open*.

eFigure 3. Comparison of Age Distribution Between Genders

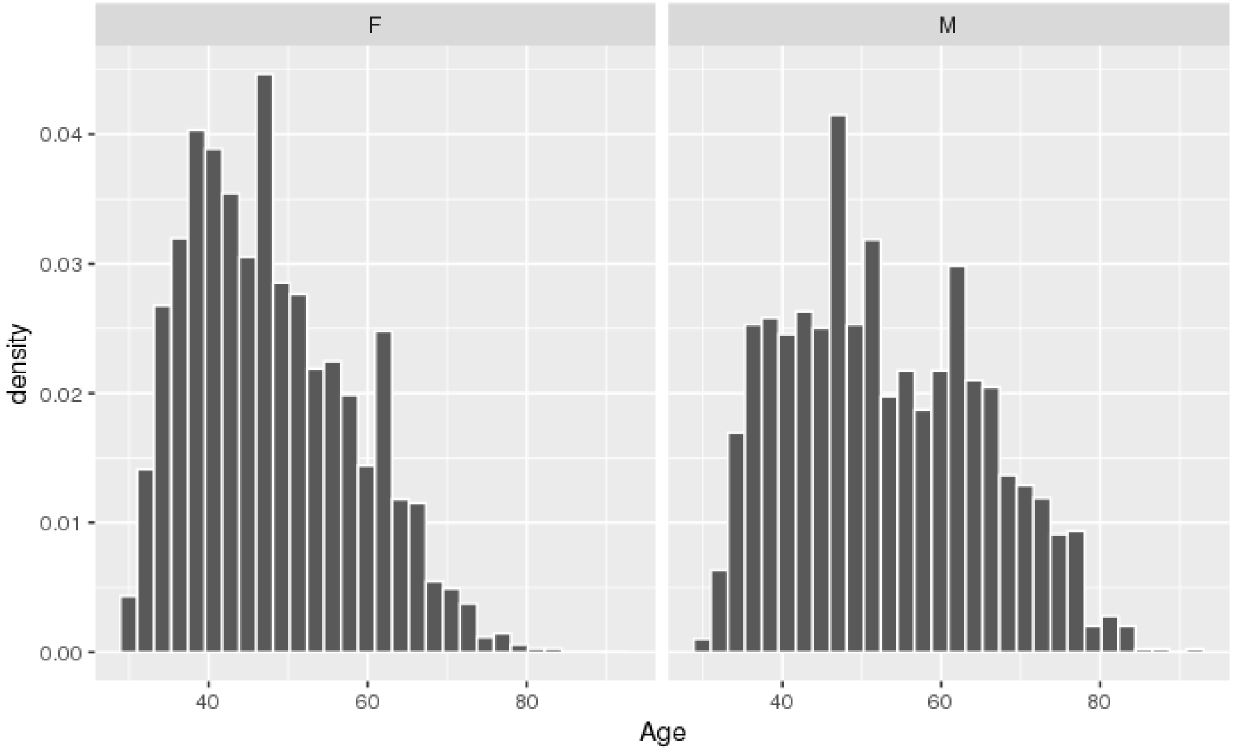

© 2021 Zachrison KS et al. *JAMA Network Open*.

eTable 1. Comparison of Innovators/Early Adopters vs Other Physicians

	Innovator or Early Adopter (N=2040)	All Others (N=1433)	Comparison across groups SMD
Characteristics of Physicians			
**Age**			
Mean (SD)	50.0 (11.0)	51.2 (12.4)	0.10
**Demographic Cohort**			0.16
Silent Gen (1928 - 1945)	29 (1.4%)	54 (3.8%)	
Boomers (1946 - 1964)	571 (28.0%)	423 (29.5%)	
Gen X (1965 - 1980)	997 (48.9%)	640 (44.7%)	
Millennials (1981 - 1996)	443 (21.7%)	316 (22.1%)	
**Gender**			0.22
F	1044 (51.2%)	580 (40.5%)	
M	996 (48.8%)	853 (59.5%)	
**Years since Medical School Graduation**			
Mean (SD)	21.8 (11.4)	22.9 (12.7)	0.09
Missing	262 (12.8%)	155 (12.5%)	
**Major Teaching Hospital affiliation**			
Yes	1338 (65.6%)	951 (66.4%)	0.02
**Specialty group**			0.46
behavioral health	177 (8.7%)	71 (5.0%)	
Primary care	599 (29.5%)	208 (14.6%)	
Medical specialty	924 (45.5%)	725 (51.0%)	

© 2021 Zachrison KS et al. *JAMA Network Open*.

	Innovator or Early Adopter (N=2040)	All Others (N=1433)	Comparison across groups SMD
surgical	331 (16.3%)	418 (29.4%)	
Missing	9 (0.4%)	11 (0.9%)	
Patient Characteristics at the Physician Level			
Mean patient volume (SD)	697.2 (534.3)	475.1 (555.4)	0.41
Mean % self-pay/Medicaid (SD)	12.0 (11.9)	12.0 (11.2)	0.00
Mean % patients 65+ (SD)	33.8 (21.5)	36.3 (22.7)	.011
Mean % non-English preferring (SD)	7.3 (8.3)	8.0 (8.2)	0.08
Mean % from a racial or ethnic minority group (SD)	21.2 (14.3)	22.2 (15.5)	0.07
Mean % with activated portal (SD)	76.5 (14.2)	70.3 (16.8)	0.39
Mean % audio-only visits (SD)	34.8 (24.6)	42.1 (32.2)*	0.25

Legend. SD – standard deviation; SMD – standardized mean differences.

* the proportion of virtual visits that were audio-only was not calculated among persistent non-adopters as these providers did not have any virtual visits

© 2021 Zachrison KS et al. *JAMA Network Open*.

eTable 2. Physician Characteristics Associated With Likelihood of Early Virtual Care Adoption (Innovator or Early Adopter Status) in Logistic Regression Modeling, Using Age in Place of Demographic Cohort Categories

	Odds Ratio	95% Confidence Interval
Age Category		
65 years and older	0.70	0.56-0.88
50-64 years	0.82	0.70-0.98
35-49 years	Ref	
Less than 35 years	0.42	0.29-0.60
Gender		
Male	Ref	
Female	1.25	1.07-1.46
Major teaching hospital affiliation	0.93	0.78-1.11
Specialty Class		
Behavioral health	3.02	2.18-4.17
Primary Care	1.78	1.43-2.21
Medical Specialty	Ref	
Surgical	0.47	0.38-0.57
Characteristics of Physicians' Patients		
Total number of patients (per 10 patient increase)	1.01	1.01-1.01
Percent of patients self-pay or Medicaid (per 5% increase)	1.04	0.99-1.10
Percent of patients over 65 (per 5% increase)	1.01	0.99-1.03
Percent of patients non-English speaking (per 5% increase)	1.08	1.01-1.17

© 2021 Zachrison KS et al. *JAMA Network Open*.

Percent of patients from a racial or ethnic minority group (per 5% increase)	0.94	0.90-0.99
Percent of patients with activated portal (per 5% increase)	1.18	1.14-1.21

© 2021 Zachrison KS et al. *JAMA Network Open*.

eTable 3. Physician Characteristics Associated With Likelihood of Early Virtual Care Adoption (Innovator or Early Adopter Status) in Logistic Regression Modeling, Including Interaction for Demographic Cohort and Specialty

	Odds Ratio	95% Confidence Interval
Demographic cohort		
Silent	0.46	0.24-0.90
Boomers	0.93	0.72-1.19
Gen X	Ref	
Millennials	1.03	0.79-1.33
Gender		
Male	Ref	
Female	1.22	1.05-1.43
Major teaching hospital affiliation	0.93	0.78-1.11
Specialty Class		
Behavioral Health	2.19	1.38-3.49
Primary Care	2.32	1.67-3.21
Medical Specialty	Ref	
Surgical	0.48	0.37-0.64
Characteristics of Physicians' Patients		
Total number of patients (per 10 patient increase)	1.01	1.01-1.01
Percent of patients self-pay or Medicaid (per 5% increase)	1.05	0.99-1.11
Percent of patients over 65 (per 5% increase)	1.01	0.99-1.04
Percent of patients non-English speaking (per 5% increase)	1.08	1.00-1.16
Percent of patients from a racial or ethnic minority group (per 5% increase)	0.94	0.90-0.99
Percent of patients with activated portal (per 5% increase)	1.18	1.14-1.21

© 2021 Zachrison KS et al. *JAMA Network Open*.

Silent Generation * Primary care	0.26	0.06-1.20
Boomers * Primary care	0.61	0.38-0.96
Millennials * Primary care	0.61	0.36-1.02
Silent Generation * Surgical	0.55	0.11-2.86
Boomers * Surgical	0.96	0.62-1.48
Millennials * Surgical	0.93	0.57-1.51
Silent Generation * Behavioral health	2.95	0.60-14.40
Boomers * Behavioral health	2.40	1.08-5.34
Millennials * Behavioral health	1.00	0.47-2.14

© 2021 Zachrison KS et al. *JAMA Network Open*.

eTable 4. Physician Characteristics Associated with Likelihood of Early Virtual Care Adoption (Innovator or Early Adopter Status) in Logistic Regression Modeling, Including Interaction Terms for Gender and Generation and for Gender and Specialty Class

	Odds Ratio	95% Confidence Interval
Demographic cohort		
Silent	0.39	0.23-0.66
Boomers	0.82	0.66-1.03
Gen X	Ref	
Millennials	0.81	0.61-1.07
Gender		
Male	Ref	
Female	1.27	0.94-1.71
Major teaching hospital affiliation	0.93	0.78-1.11
Specialty Class		
Behavioral Health	2.43	1.59-3.70
Primary Care	1.74	1.28-2.37
Medical Specialty	Ref	
Surgical	0.51	0.40-0.65
Characteristics of Physicians' Patients		
Total number of patients (per 10 patient increase)	1.01	1.01-1.01
Percent of patients self-pay or Medicaid (per 5% increase)	1.05	0.99-1.11
Percent of patients over 65 (per 5% increase)	1.01	0.99-1.03
Percent of patients non-English speaking (per 5% increase)	1.09	1.01-1.17
Percent of patients from a racial or ethnic minority group (per 5% increase)	0.94	0.90-0.98
Percent of patients with activated portal (per 5% increase)	1.18	1.14-1.21

© 2021 Zachrison KS et al. *JAMA Network Open*.

Silent Generation * Female	0.97	0.21-4.55
Boomers * Female	1.14	0.79-1.64
Millennials * Female	1.27	0.87-1.86
Primary Care * Female	0.95	0.63-1.44
Surgical * Female	0.79	0.53-1.18
Behavioral health * Female	1.54	0.81-2.94
Total number of patients * Female	1.00	1.00-1.00

© 2021 Zachrison KS et al. *JAMA Network Open*.

eTable 5. Physician Characteristics Associated with Likelihood of Persistent Nonadoption of Virtual Care in Logistic Regression Modeling

	Odds Ratio	95% Confidence Interval
Demographic cohort		
Silent	0.82	0.32-2.12
Boomers	1.12	0.77-1.62
Gen X	Ref	
Millennials	0.61	0.39-0.97
Female gender	0.67	0.47-0.95
Major teaching hospital affiliation	0.51	0.35-0.73
Specialty Class		
Behavioral Health	0.02	0.01-0.10
Primary Care	0.54	0.31-0.96
Medical Specialty	Ref	
Surgical	1.13	0.76-1.67
Characteristics of Physicians' Patients		
Total number of patients (per 10 patient increase)	0.97	0.96-0.98
Percent of patients self-pay or Medicaid (per 5% increase)	1.02	0.93-1.12
Percent of patients over 65 (per 5% increase)	0.96	0.92-1.001
Percent of patients non-English speaking (per 5% increase)	0.92	0.81-1.05

© 2021 Zachrison KS et al. *JAMA Network Open*.

Percent of patients from a racial or ethnic minority group (per 5% increase)	0.98	0.90-1.05
Percent of patients with activated portal (per 5% increase)	0.77	0.74-0.81

© 2021 Zachrison KS et al. *JAMA Network Open*.
